# Clinical Features of High-Grade Extremity and Trunk Sarcomas in Patients Aged 80 Years and Older: Why Are Outcomes Inferior?

**DOI:** 10.3389/fsurg.2019.00029

**Published:** 2019-05-31

**Authors:** Jungo Imanishi, Lester W. M. Chan, Matthew L. Broadhead, Grant Pang, Samuel Y. Ngan, John Slavin, Stephen Sharp, Peter F. M. Choong

**Affiliations:** ^1^Department of Orthopedics, St. Vincent's Hospital, Fitzroy, VIC, Australia; ^2^Department of Orthopedic Oncology and Surgery, Saitama Medical University International Medical Center, Hidaka, Japan; ^3^Department of Orthopaedic Surgery, Saitama Medical University Hospital, Moroyama, Japan; ^4^Department of Orthopaedic Surgery, Tan Tock Seng Hospital, Singapore, Singapore; ^5^Bone and Soft Tissue Tumour Unit, Peter MacCallum Cancer Centre, Melbourne, VIC, Australia; ^6^Division of Radiation Oncology, Peter MacCallum Cancer Centre, Melbourne, VIC, Australia; ^7^Department of Pathology, St. Vincent's Hospital, Fitzroy, VIC, Australia; ^8^Department of Surgery, University of Melbourne, Melbourne, VIC, Australia

**Keywords:** soft tissue sarcoma, geriatric patients, inferior prognosis, surgery, local recurrence, metastasis, tumor biology, metastasectomy

## Abstract

**Background:** The population of many countries is aging and a significant number of elderly patients with soft-tissue sarcoma are being seen at cancer centers. The unique therapeutic and prognostic implications of treating soft-tissue sarcoma in geriatric patients warrant further consideration in order to optimize outcomes.

**Patients and Methods:** This is a single-institution retrospective study of consecutive non-metastatic primary extremity and trunk high-grade sarcomas surgically treated between 1996 and 2012, with at least 2 years of follow-up for survivors. Patient characteristics and oncological outcomes were compared between age groups (≥80 vs. <80 years), using Chi-square or Fisher-exact test and Log-Rank or Wilcoxon test, respectively. Deaths from other causes were censored for disease-specific survival estimation. A p< 0.05 was regarded as statistically significant.

**Results:** A total of 333 cases were eligible for this study. Thirty-six patients (11%) were aged ≥80 years. Unplanned surgery incidence and surgical margin status were comparable between the age groups. Five-year local-recurrence-free, metastasis-free and disease-specific survivals were 72% (≥80 years) vs. 90% (<80 years) (*p* = 0.004), 59 vs. 70% (*p* = 0.07) and 55 vs. 80% (*p* < 0.001), respectively. A significantly earlier first metastasis after surgery (8.3 months vs. 20.5 months, mean) and poorer survival after first metastasis (*p* = 0.03) were observed. Cox analysis revealed “age ≥80 years” as an independent risk factor for local failure and disease-specific mortality, with hazard ratios of 2.41 (95% CI: 1.09–5.32) and 2.52 (1.33–4.13), respectively. A competing risks analysis also showed that “age ≥80 years” was significantly associated with the disease-specific mortality.

**Conclusions:** Oncological outcomes were significantly worse in high-grade sarcoma patients aged ≥80 years. The findings of more frequent local failure regardless of a consistent primary treatment strategy, an earlier time to first metastasis after surgery, and poorer prognosis after first metastasis suggest that more aggressive tumor biology, in addition to multiple co-morbidity, may explain the inferiority.

## Introduction

Longevity has increased dramatically in many developed countries. Australia enjoys one of the longest life expectancies in the world; in 2017, nearly 4% of the Australian population was 80 years or older (1). In treating elderly patients, it is important to consider the unique therapeutic and prognostic implications of advancing age.

Soft-tissue sarcomas (STSs) are a group of rare malignant neoplasms of mesenchymal origin. The incidence is approximately 3 per 100,000 population per year (2). Although typically recognized as a disease of middle age, many STSs are diagnosed in elderly patients (3). The mainstay of treatment for STS is surgery with or without adjuvant radiotherapy (4). Adjuvant chemotherapy is reserved for chemotherapy-sensitive subtypes such as primitive neuroendocrine tumor and alveolar/embryonal rhabdomyosarcoma. The indication for other histotypes is controversial.

Inferior clinical outcomes for older sarcoma patients have been reported by previous authors. Al-Refaie et al. reported an association between older age and higher disease-specific mortality in a large US population-based study, but, details of treatment, local recurrence or metastasis in the elderly population were not available (5). Biau et al. conducted a multicenter retrospective study and reported more frequent positive margins for older patients and an adverse effect of older age on prognosis (6). The reasons for inferior outcomes in the elderly, however, remain unanswered.

Further studies are required to evaluate the impact of increasing age on prognosis of STSs and investigate the reasons for the reported inferior prognosis in elderly patients. At our center, patients with STS have been managed with the same treatment and follow-up protocols regardless of age for the last two decades. Treatment consists of neo-adjuvant radiotherapy and surgical resection. The aim of this study was to compare the clinical features and oncological outcomes of elderly patients with a younger population using a local treatment strategy that does not stratify for age. Secondly, we aimed to assess possible factors that could account for differences in outcomes between age groups.

## Patients and Methods

This is a single-institution retrospective study of consecutive cases. Institutional review board approval was obtained prior to the study (HREC number: QA 006/16), and the study was conducted in accordance with the Helsinki declaration 2013. Subjects were identified from the institutional surgical database. Medical records were reviewed and updated with oncological status information from the state cancer council. Patients were categorized into two age groups (<80 and ≥80 years). The age cut off was decided before analysis and based upon the precedent set by previous studies (3, 5, 7).

Inclusion criteria were patients with primary extremity or trunk high-grade STSs, surgically treated between 1996 and 2012 with ≥2 years of follow-up for survivors. Patients with lymph node or distant metastases at time of surgery (*n* = 40, two cases were aged ≥80 years) were excluded. Patients undergoing re-excision after unplanned sarcoma excision were included. Intermediate malignancy tumors, such as atypical lipomatous tumor and solitary fibrous tumor, and grade 1 sarcomas according to the Fédération Nationale des Centers de Lutte le Cancer (FNCLCC) grading system were not included in this study because of their low potential for metastasis and mortality (8, 9).

Pre-operative radiotherapy to a total dose of 50.4 Gy, was delivered by external beam over 28 sessions for the majority of the cases except for STSs for which amputation was planned and achievable with wide margins at the first presentation. The indication for chemotherapy was determined at the weekly institutional multi-disciplinary meeting based on the histotype, age and general condition of the patient. Surgery was performed at 3 to 8 weeks after the completion of radiotherapy, aiming for microscopically negative margins unless the tumor abutted vital structures such as major blood vessels or nerves.

After surgery, the specimen was examined macroscopically and microscopically by pathologists and reviewed at the regular weekly multi-disciplinary meeting. The institutional post-operative follow-up protocol included regular clinical review every 3 months for the first 2 years, 6 monthly for the next 2 years, and then yearly for the next 4 years for a total of 8 years continuous follow-up. Computed tomography (CT) examination of the lungs and magnetic resonance imaging (MRI) of the affected part would be undertaken regularly during this follow-up period.

Patient age was determined as the age when the pathological diagnosis was confirmed. Tumor size was measured as the maximum diameter of tumor mass on any axis from MRI or CT at first presentation prior to any treatment. For unplanned excision cases, if both MRI and CT were unavailable, the size was estimated based on pathological reports, referral letters and surgical scars. Pathological diagnoses were retrieved from medical records. Malignant fibrous histiocytomas and cases with unclear diagnoses, such as “myxoid sarcoma” and “high-grade sarcoma”, were reviewed and re-classified according to the World Health Organization (WHO) classification in 2013 (10). The FNCLCC grading system was used for tumor grading. With the aforementioned factors, all patients were staged using the 7th edition of the American Joint Committee on Cancer (AJCC) staging system (11). A tumor partially or entirely deep to or engaged in the deep fascia was classified as deep. Margins were classified as positive or negative based on pathology reports. In cases where margin evaluation was difficult due to post-radiation reaction, wide and marginal margins according to the Enneking system were considered as microscopically negative, while intralesional as positive (12), because a marginal margin with pre-operative radiotherapy is considered to be equivalent to a wide margin without adjuvant radiotherapy (13).

The local-recurrence-free survival (LRFS) and metastasis-free survival (MFS) time were calculated from the date of surgery to the date of recurrence at or near the primary tumor site and metastasis at any other site, respectively. For these two survival time points, the date of tissue biopsy of recurrence or metastasis was applied if the diagnosis was pathologically confirmed, otherwise the date of radiologic study detecting local recurrence or metastasis was applied for LRFS and MFS. The disease-specific survival (DSS) and overall survival (OS) were calculated from the date of diagnosis to the date of death. DSS after first metastasis was counted from the date of first metastasis for advanced cases only.

## Statistical Analysis

Differences in categorical data were analyzed using Chi-square test or Fisher-exact test. LRFS, MFS, DSS, and OS probabilities, were calculated and compared using Kaplan-Meier method and Log-rank or Wilcoxon test. In this study, Wilcoxon test was only used for DSS after first metastasis to more effectively detect disease-specific mortality at an earlier stage. Possible prognostic variables chosen for univariate Log-rank test and Cox proportional hazard multivariate analysis were patient gender, age, tumor location, depth, AJCC stage, surgical margin, adjuvant radiotherapy, adjuvant chemotherapy, and presence of previous unplanned excision. Depth was chosen because it is not included in the current AJCC staging system. A *p* < 0.05 was regarded as statistically significant. All statistical analyses were computed using SPSS version 17 (SPSS Inc., Chicago, USA).

In this study, oncological status “died of other disease (DOOD) ” can be a competing risk factor against “died of disease (DOD),” which was expected to be more frequent and have more impact among the older age group. A competing risks analysis and regression modeling of competing risk, using R, were also conducted. R 3.5.3 application, its packages or function (“cmprsk”, “CumIncidence” and “crr-addson”), were installed online and used for competing risks analysis (14–17).

## Results

Three-hundred and 33 surgically-treated extremity or trunk high-grade sarcomas were included in this study. Thirty-six (11%) were aged ≥80 years. The male: female ratio was 1.3: 1. The age range was 15–95 (mean, 55) years. Follow-up period for survivors ranged 24–206 (mean, 83) months.

Tumors were located in 298 extremities and 35 trunk walls. The most common histotype was undifferentiated pleomorphic sarcoma (UPS) (133 cases, 40%), followed by myxoid liposarcoma (45 cases, 14%), synovial sarcoma (39 cases, 12%), leiomyosarcoma (30 cases, 9%), and myxofibrosarcoma (29 cases, 9%). The tumor size ranged 1–37 (mean, 8.2) cm, with 208 cases (62%) being >5 cm in maximum diameter. Eighty-six sarcomas (26%) were superficial. There were 180 AJCC stage II and 153 stage III sarcomas.

### Difference in Background Among the Age Groups

There were significant differences between the age groups for histologic subtype (*p* = 0.0028). For patients aged ≥80 years UPS was more common, whereas myxoid liposarcoma and synovial sarcoma were less common (Table 1). AJCC stage tended to be higher for the older group, but the *p*-value was just over 0.05. No significant difference was observed in the tumor size (*p* = 0.2004), the surgical margin status (*p* = 0.2635) or the presence of previous unplanned excision (p = 0.8105).

**Table 1:** Characteristics of sarcoma patients aged ≥80 years and <80 years.

**Characteristic**		**Age ≥ 80**	**Age < 80**	***p*-value**
		**No. (percent)**	**No. (percent)**	
Total		36	297^**a**^	
Gender	Male	23 (64%)	166 (56%)	0.3604^b^
Site	Trunk	5 (14%)	30 (10%)	0.4840^b^
Unplanned surgery	(+)	12 (33%)	105 (35%)	0.8105^b^
**Histotype**^**a**^	**UPS**	**23 (64%)**	**110 (37%)**	0.0028^*^^b^
	**MLPS**	**1 (3%)**	**44 (15%)**	
	**SS**	**1 (3%)**	**38 (13%)**	
	**LMS**	**3 (8%)**	**27 (9%)**	
	**MFS**	**6 (17%)**	**23 (8%)**	
	**Others**	**2 (6%)**	**55 (19%)**	
Size	≤5 cm	10 (28%)	115 (39%)	0.2004^b^
Depth	Superficial	9 (25%)	77 (26%)	0.9046^b^
AJCC stage	IIA+IIB	14 (39%)	166 (50%)	0.0532^b^
	III	22 (61%)	131 (40%)	
Amputation	(+)	3 (8%)	26 (9%)	1^d^
Surgical Margin	Positive	4 (11%)	17 (6%)	0.2635^c^
	Negative	32 (89%)	280 (94%)	
Adjuvant RT	(+)	32 (89%)	262 (88%)	0.5469^c^
Adjuvant CT	(+)	0	20 (7%)	0.0944^d^

*UPS, undifferentiated pleomorphic sarcoma; MLPS, myxoid liposarcoma; SS, synovial sarcoma; LMS, leiomyosarcoma; AJCC, American Joint Committee on Cancer; RT, radiotherapy; CT, chemotherapy*.

An asterisk (

* *) in bold means a significant difference (p < 0.05)*.

a *Six children aged <18 years were included. Most common hsitotype was synovial sarcoma (n = 3), follow by clear cell sarcoma, myxoid liposarcoma, and extra-renal rhabdoid tumor (one each)*.

b *Chi-square test*.

c *Two-sided Fisher-exact test*.

d *One-sided Fisher-exact test assuming less frequent administration for the older group*.

### Survival Analysis

**Figure 1:**
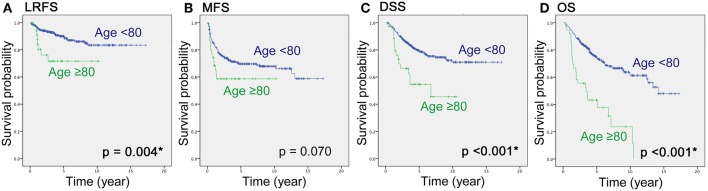
Local-recurrence-free survival **(A)**, metastasis-free survival **(B)**, disease-specific survival **(C)** and overall survival **(D)** of high-grade sarcomas in patients aged <80 and ≥80 years, using the Kaplan-Meier method. *LRFS, local-recurrence-free survival; MFS, metastasis-free survival; DSS, disease-specific survival; OS, overall survival*.

RFS, MFS, DSS and OS were compared between the two age groups by the Kaplan-Meier method. There were significant differences in LRFS, DSS and OS between those aged <80 and ≥80 years (Figure 1A, *p* = 0.004; Figure 1C, *p* < 0.001; Figure 1D, *p* <0.001), but a trend toward worse MFS for the older group (Figure 1B, *p* = 0.07) (Table 2).

**Table 2:** Comparison of oncologic outcomes between age groups using the Kaplan-Meier method.

**Oncological outcome**	**Age <80 (95% CI)**	**Age ≥80 (95% CI)**	***p*-value**
5-year LRFS			
**All**	**90.2 (86.5–93.9)%**	**71.7 (54.6–88.8)%**	0.003^*^^a^
**Stage IIA+IIB**	**94.5 (90.8–98.2)%**	**77.9 (55.8–100)%**	0.013^*^^a^
Stage III	83.5 (76.0–91.0)%	67.8 (43.9–91.7)%	0.172**^a^**
5-year MFS			
All	69.6 (64.1–75.1)%	58.6 (41.9–75.3)%	0.070**^a^**
Stage IIA+IIB	79.8 (73.3–86.3)%	62.9 (36.8–89.0)%	0.055**^a^**
Stage III	56.1 (47.3–64.9)%	55.8 (34.0–77.6)%	0.721**^a^**
5-year DSS			
**All**	**79.6 (74.7–84.5)%**	**54.6 (36.6–72.6)%**	<0.001^*^^a^
**Stage IIA+IIB**	**87.9 (82.4–93.4)%**	**64.3 (35.1–93.5)%**	0.032^*^^a^
**Stage III**	**68.9 (60.7–77.1)%**	**47.4 (24.7–70.1)%**	0.026^*^^a^
5-year OS			
**All**	**75.0 (69.7–80.3)%**	**43.2 (26.7–59.7)%**	<0.001^*^^a^
**Stage IIA+IIB**	**84.8 (78.7–90.9)%**	**53.6 (25.8–81.4)%**	<0.001^*^^a^
**Stage III**	**62.5 (53.9–71.1)%**	**36.4 (16.2–56.6)%**	0.001^*^^a^
1-year DSS after first metastasis			
**All**	**72.0 (62.6–81.4)%**	**35.7 (10.6–60.8)%**	0.034^*^^b^

*CI, confidence interval; LRFS, local-recurrence-free survival; MFS, metastasis-free survival; DSS, disease-specific survival; Met, metastasis*.

An asterisk (

* *) in bold means a significant difference (p < 0.05)*.

a *Log-Rank test*.

b *Wilcoxon test*.

**Figure 2:**
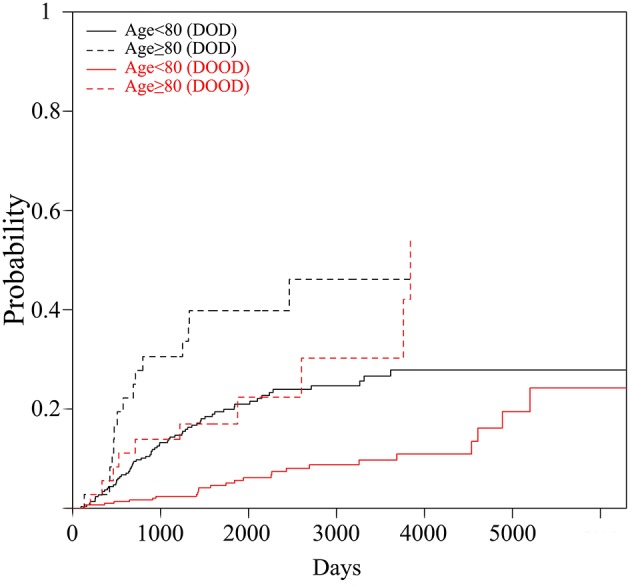
The graph shows cumulative incidence estimation of the probability of the occurrence of DOOD and DOD by the two age groups (Age <80 vs. Age≥80).

Regarding patient survival, the ratio of those that died of other disease (DOOD) was significantly higher in the elderly group (10/36 over 25/297), therefore a competing analysis using R was added. Both DOD and DOOD were significantly more frequent among the older group with *p*-values of 0.0061 and <0.0001 (Figure 2). The estimated incidence was 10% (<80 years) vs. 28% (≥80 years) at 2 years and 20 vs. 40% at 5 years for DOD (*p* = 0.006), and 2% (<80 years) vs. 14% (≥80 years) at 2 years and 5% vs. 17% at 5 years for DOOD (*p* < 0.001).

More frequent life-threatening local recurrence for the geriatric population was thought to be a possible factor affecting disease-specific morbidity because a significant difference in DSS was observed despite a non-significant difference in MFS. The latest oncological status was reviewed for patients developing local recurrence. Thirty-two of 297 patients aged <80 years and eight of 36 of those aged ≥80 years developed local recurrence. Among these 40 recurrent cases, six patients (19%) aged <80 years and two (25%) of those aged ≥80 years eventually died of local recurrence without any metastasis.

Ninety of 297 STS patients aged <80 years developed first metastasis 1–156 (mean; 20.5) months after surgery, whereas 14 of 36 those aged ≥80 years 2–16 (mean; 8.3) months post-operatively. Treatments for metastasis, including surgery, chemotherapy and radiotherapy, and DSS after first metastasis were compared between the two age groups because different treatment strategies for advanced conditions can affect overall DSS. The administration rate of any of the aforementioned treatments was significantly higher for the younger group aged <80 years (6/14 vs. 80/90, *p* < 0.001). Of note, metastasectomy was less often performed in patients aged ≥80 years (4/14 vs. 55/90, *p* = 0.0223) (Table 3).

**Table 3:** Administration rate of treatment for metastases between age groups.

	**Age <80 years**	**Age ≥80 years**
Treatment for Metastasis		
any	6/14 (43%)	80/90 (89%)
Surgery	4/14 (29%)	55/90 (61%)
Chemotherapy	0/14 (0%)	21/90 (23%)
Radiotherapy	2/14 (14%)	27/90 (30%)

**Figure 3:**
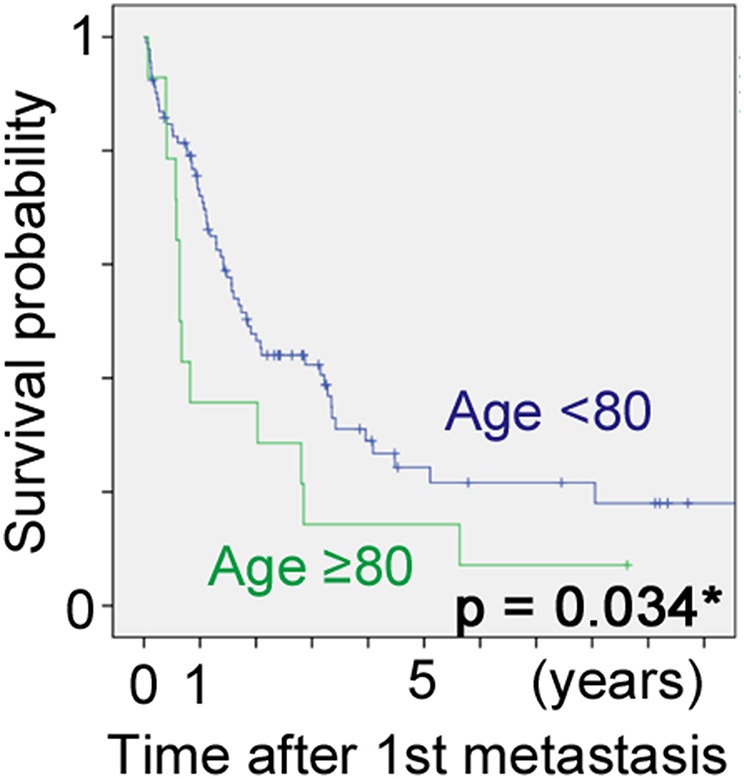
Disease-specific survival after first metastasis of high-grade sarcomas in patients aged <80 and ≥80 years, using the Kaplan-Meier method.

DSS after first metastasis was significantly worse for the elderly group, with 1-year and 5-year probabilities of 72 vs. 36% and 24 vs. 14%, respectively (Wilcoxon test: *p* = 0.03, Figure 3).

### Prognostic Factor Analysis

Significant factors on univariate analysis were margin status (*p* = 0.003), age (*p* = 0.004) and AJCC stage (*p* = 0.014) for LRFS, AJCC stage (*p* < 0.001), and depth (*p* = 0.001) for MFS, and AJCC stage (*p* < 0.001), age (*p* < 0.001), and depth (*p* = 0.032) for DSS. Cox analysis using the aforementioned factors revealed “age ≥80 years” as an independent predictor for lower LRFS and DSS along with AJCC stage 3, with hazard ratio of 2.41 (95% confidence interval (CI): 1.09–5.32) and 2.34 (95% CI: 1.33–4.13), respectively (Table 4).

**Table 4:** Univariate and multivariate analyses of prognostic factors for survivals.

**Univariate analysis**			**Multivariate Cox analysis**	
**Variables**		***p*-value**	**HR (95% CI)**	***p*-value**
**LRFS**				
**Margin**	Positive	0.003	**3.09 (1.35–7.04)**	0.007^*^
	Negative		**Reference**	
**AJCC Stage**	IIA	0.014	**0.39 (0.19–0.81)**	0.024^*^
	IIB		**0.45 (0.17–1.18)**	
	III		**Reference**	
**Age**	<80 years	0.004	**Reference**	0.029^*^
	≥80 years		**2.41 (1.09–5.32)**	
**MFS**				
**AJCC stage**	IIA	< 0.001	**0.40 (0.25–0.64)**	< 0.001^*^
	IIB		**0.47 (0.26–0.83)**	
	III		**Reference**	
**Depth**	Superficial	0.001	**Reference**	0.027^*^
	Deep		**1.89 (1.08–3.33)**	
**DSS**				
**AJCC stage**	IIA	< 0.001	**0.35 (0.19–0.61)**	< 0.001^*^
	IIB		**0.44 (0.23–0.88)**	
	III		**Reference**	
**Age**	<80 years	< 0.001	**Reference**	0.003^*^
	≥80 years		**2.34 (1.33–4.13)**	
Depth	Superficial	0.032		0.231
	Deep			

*LRFS, local-recurrence-free survival; MFS, metastasis-free survival; DSS, disease-specific survival; HR, hazard ratio; CI, confidence interval*.

An asterisk (

* *) in bold means a significant difference (p < 0.05)*.

The same dataset was analyzed using the competing risks analysis. Selected factors for analysis were age, gender, tumor location, AJCC stage, previous unplanned excision, adjuvant chemotherapy, adjuvant radiotherapy, surgical margin. Competing risk regression model revealed the age 80 years and the higher AJCC stage as significant factors affecting DOD, and the sub-distribution hazard ration, considering DOOD as a competing risk factor, was 1.99 (95% CI: 1.09–3.61) for age ≥80 years and 2.68 (95% CI: 1.66–4.34) for AJCC stage III (Table 5).

**Table 5:** Competing risk regression analysis using R for disease-specific mortality.

**Factors**		**Reference**	**Exp (coef) [95% CI]**	**p-value**
**Age**	**≥80 years**	** <80 years**	**1.99 [1.09–3.61]**	0.025^*^
Gender	Female	Male	1.076	0.76
Tumor location	Trunk	Extremity	1.167	0.67
**AJCC stage**	**Stage III**	**Stage IIA** **+** **IIB**	**2.68 [1.66–4.34]**	<0.001^*^
Previous UPE	Previous UPE	No previous UPE	1.215	0.43
Adjuvant CT	Adjuvant CT	No adjuvant CT	1.330	0.62
Adjuvant RT	Adjuvant RT	No adjuvant RT	0.987	0.98
Margin status	Positive	Negative	1.399	0.34

*CI, confidence interval; UPE, unplanned excision; CT, chemotherapy; RT, radiotherapy*.

An asterisk (

* *) in bold means a significant difference (p < 0.05)*.

## Discussion

This study examined the clinical features of sarcomas in patients aged 80 years or older. Despite similar treatment quality for initial treatment, including previous unplanned excision rate and margin status, oncological outcomes in the older population were significantly poorer. Regarding disease-specific survival, however, one statistic concern arises. Frequent death in the elderly from causes unrelated to STS may result in an overestimation of DSS in this group. In this study, 10 (28%) out of 36 STS patients aged ≥80 years died of other disease. If the frequency of competing events exceeds 10–20%, the use of alternatives to Kaplan-Meier survivorship is recommended (18). In this study, along with the Kaplan-Meier method, the competing risks analysis using R showed the two-fold high risk of DOD for the age of ≥80 years.

Our data suggest that this inferiority could be attributed to different histotype distribution leading to more aggressive tumor biology and manifesting itself in higher local recurrence rates and poorer prognosis after metastasis. The aforementioned results are consistent with previous reports from Al-Refaie et al. (5), Biau et al. (6), Buchner et al. (19) and Lahat et al. (20). The first two papers postulated that the inferior prognosis could be due to under-treatment in the elderly population although they were unable to demonstrate this from their data. In our study, the primary treatment quality was similar between the age groups, with similar surgical margin quality and previous unplanned excision incidence of around 35%. As such, our data does not suggest initial under-treatment as a main reason for the inferior prognosis in older sarcoma patients.

In this study, MFS was not significantly different (*p* = 0.07), however, DSS was significantly lower for patients aged ≥80 years (*p* < 0.001). DSS is usually considered to be predominantly affected by metastatic disease. The discordance between MFS and DSS suggests that DSS may be affected by other factors such as local control or less aggressive of efficacious management of metastatic disease in the older age group

Poor local control of sarcomas in patients aged ≥80 years has been reported in previous studies. In 2006, Boden et al. reported a local recurrence rate of 22% for surgically treated sarcoma patients aged >80 years with a mean follow-up of 22 months (*n* = 50) (3). In 2014, a multi-institutional study conducted reported a 5-year LRFS of 52% for sarcoma patients aged ≥80 (*n* = 29) (7). The 5-year LRFS of 72% for those aged ≥80 (*n* = 36) in our study does not contradict these reports. Considering the relatively high ratio (89%) of negative surgical margin achieved in our cohort, local behavior of STSs in the elderly patients may be more aggressive than for younger patients. Of note, the impact of local control on mortality cannot be ignored; indeed, in our study, two patients aged ≥80 years died of direct tumor invasion into vital structures. Improved local control may be crucial for improving the prognosis of sarcoma patients aged ≥80 years, especially for sarcomas arising in the trunk or proximal part of the extremity.

In this cohort, DSS after first metastasis was significantly worse for patients aged ≥80 years (*p* = 0.03). Possible explanations for this include patient factors, disease factors and treatment factors. We observed that patients ≥80 years were less likely to undergo treatment for metastases. This included radiotherapy, chemotherapy and surgery. Administration ratio of any kind of treatment for metastases was 6/14 (≥80 years) vs. 80/90 (<80 years). Of note, metastasectomy was significantly less frequently performed among the geriatric patients, 4/14 (≥80 years) vs. 55/90 (<80 years). Although the role of metastasectomy for STS is still under investigation, many studies have shown fair clinical results of pulmonary metastasectomy for selected metastatic STSs, with up to 50% 5-year survival reported after pulmonary resection (21, 22). Less aggressive treatment toward geriatric sarcoma patients may be a contributing factor for inferior patient survival.

The increased mortality for sarcoma patients aged ≥80 years may also be explained by factors specific to the geriatric population, including multiple co-morbidities, reduced physiological reserve, polypharmacy, and physician or family treatment bias for under-treatment (23). Age-related changes in tumor biology and host response may also be contributory and should be considered. It was previously believed that tumors in the elderly are less aggressive and metastasize less readily (24), however, both epidemiological and experimental studies suggest that this may not be the case. For instance, fibrosarcomas induced by 3-methylcholanthrene and UV-light induced sarcomas grow more rapidly in older animals compared to young animals and this may be related to an age-related decline in cytotoxic T-cell function (25, 26). It has also been suggested that tumors that are more aggressive in older animals are highly immunogenic, being chemically or virally induced, and that age-related decline in immune function leads to more aggressive disease (24). In this study, the occurrence of first metastasis was earlier in the age ≥80 group compared to the age <80 group (mean, 8.3 vs. 20.5 months); we postulate that this may reflect more aggressive tumor behavior in the elderly population.

This study has several limitations. Firstly, the retrospective study design with a relatively small number of patients in the elderly group and a relatively short follow-up period to evaluate late disease-specific mortality difference. Secondly, the ambiguity of judgement of DOD or DOOD. Judgement of DOD or DOOD by a clinician may not be replicable. Thirdly is the difficulty in accurately assessing margin status. Pathological examination of tumor borders and viability become difficult after neo-adjuvant radiotherapy. Lastly, although there was no significant difference in initial surgical treatment quality, significantly less metastasectomy or chemotherapy was attempted for the age ≥80 years group. Not only the administration rate, but dose intensity is usually reduced for the older patients aged ≥80 years. This should be regarded as a confounding factor for the analysis of DSS and OS.

## Conclusions

DSS of high-grade STS in patients aged ≥80 years is shorter than for younger counterparts despite the same primary treatment strategy. Possible contributing factors include a worse local control and shorter survival after first metastasis. Poorer survival after first metastasis may be due to more aggressive tumor biology or less aggressive treatment of advanced disease in elderly patients. More aggressive treatment may not be feasible for elderly patients and it is uncertain whether it would improve their outcomes; however, less aggressive treatment is not recommended from an oncological perspective.

## Ethics Statement

This study was carried out in accordance with the recommendations of the National Statement on the Ethical Human research (NHMRC; 2007). Written and informed consent was not required. The protocol was approved by the Human Research Ethics Committee (HREC)-A, St. Vincent's Hospital, Melbourne (HREC number: QA 006/16).

## Author Contributions

JI contributed to the study protocol, collected and analyzed data, conducted literature research, and prepared the manuscript. LC assisted in the study design and preparation of the manuscript. MB helped the data analysis and added a part of Discussion. GP, SN, JS, and SS contributed to study design and assisted in preparation of the manuscript. PC provided the idea of this study, contributed to the study protocol, and edited the manuscript.

### Conflict of Interest Statement

The authors declare that the research was conducted in the absence of any commercial or financial relationships that could be construed as a potential conflict of interest.
